# Budesonide treatment for microscopic colitis from immune checkpoint inhibitors

**DOI:** 10.1186/s40425-019-0756-0

**Published:** 2019-11-07

**Authors:** Michael S. Hughes, Gabriel E. Molina, Steven T. Chen, Hui Zheng, Vikram Deshpande, Riley Fadden, Ryan J. Sullivan, Michael Dougan

**Affiliations:** 1 Harvard Medical School, Boston, MA USA; 2 Present address: Johns Hopkins Medical Institutions, Baltimore, MD USA; 3 Department of Dermatology, Massachusetts General Hospital, Boston, MA USA; 4 Department of Medicine, Massachusetts General Hospital, Boston, MA USA; 5 Biostatistics Center, Massachusetts General Hospital, Boston, MA USA; 6 Department of Pathology, Massachusetts General Hospital, Boston, MA USA; 7 Division of Oncology, Massachusetts General Hospital, Boston, MA USA; 8 Division of Gastroenterology, Massachusetts General Hospital, Boston, MA USA

## Abstract

**Background:**

Immune checkpoint inhibitors (CPIs) are effective against a variety of malignancies but can be limited by inflammatory toxicities such as enterocolitis. Enterocolitis is typically treated with systemically active glucocorticoids. Endoscopy can stratify patients by the severity of mucosal inflammation, including identifying patients with colitis in the absence of visible mucosal changes: microscopic colitis. Whether patients with CPI microscopic colitis could be managed differently from colitis with more severe mucosal involvement is unclear. The objective of this study was to describe outcomes in CPI microscopic colitis focusing on the response to first line treatment with budesonide.

**Methods:**

We evaluated data from a retrospective cohort from a single-center large academic hospital. The participants were all adult patients evaluated by endoscopy for suspected CPI enterocolitis between 3/2017 and 3/2019. The exposures were: Mayo Endoscopic Score (range 0–3). The subset was: oral budesonide, maximum dose 12 mg daily, administered minimum of 5 weeks. The main outcomes and measures were: Primary: time from first CPI exposure to first glucocorticoid use; use of systemic glucocorticoids; time from symptom onset to resolution; continuation of CPI therapy; number of additional CPI infusions received. Secondary: admissions for symptom control; novel irAE development; need for second-line immunosuppression; oncologic outcomes.

**Results:**

We identified 38 patients with biopsy confirmed CPI enterocolitis, 13 in the microscopic colitis cohort, and 25 in the non-microscopic colitis cohort. Budesonide use was higher in the microscopic colitis cohort (12/13 vs 3/25, *p* < 0.001), and systemic glucocorticoid use was higher in non-microscopic colitis (22/25 vs. 3/13, *p* < 0.001). Time from symptom onset to resolution did not differ. Microscopic colitis patients more frequently remained on CPI after developing (entero)colitis (76.9% vs 16.0%, *p* < 0.001). Microscopic colitis patients tolerating further CPI received, on average, 4.2 CPI infusions more than non-microscopic colitis patients tolerating CPI (5.8 vs 1.6, *p* = 0.03). Microscopic colitis was associated with increased time-to-treatment-failure (HR 0.30, 95% CI 0.14–0.66) and progression-free survival (HR 0.22, 95% CI 0.07–0.70).

**Conclusions:**

Gastrointestinal mucosal inflammation without visible mucosal injury is a distinct, prevalent CPI enterocolitis subset that can be diagnosed by endoscopy. First-line budesonide appears effective in controlling “microscopic colitis” symptoms and prolonging immunotherapy duration. These findings present a compelling rationale for routine endoscopic evaluation of suspected CPI enterocolitis and suggest an alternative glucocorticoid-sparing treatment strategy for a subset of such patients.

## Introduction

Immune checkpoint inhibitors (CPIs) are highly effective against a range of advanced malignancies, but are also associated with treatment-limiting inflammatory toxicities termed “immune-related adverse events” (irAEs) [1–5]. IrAEs can involve any organ system, although toxicities involving barrier organs are the most common [1, 2, 6]. The spectrum and severity of irAEs are related to the specific checkpoint pathway inhibited, with cytotoxic T-lymphocyte antigen-4 (CTLA-4) inhibitors generally associated with more frequent and more severe irAEs compared to inhibitors of programmed cell death-1 (PD-1) or its ligand (PD-L1); combination immunotherapies are associated with the highest rates of toxicity, and are likely to see increased clinical use in the future [1, 2, 6]. We are beginning to understand the predictors of treatment response to CPIs, yet our understanding of the causes and predictors of irAEs, as well as optimal diagnostic and management strategies, is substantially more limited [1, 2, 7–9].

(Entero)colitis is among the most common and severe irAEs associated with current CPIs, and is an important reason for CPI discontinuation, particularly in patients treated with combination immunotherapy blocking both PD-1 and CTLA-4 [1, 6, 10–12]. CPI enterocolitis typically responds to systemic glucocorticoids, with a smaller proportion of patients requiring secondary immune suppression using the tumor necrosis factor alpha inhibitor infliximab or the integrin inhibitor vedolizumab [6, 12–16]. Although effective at resolving many irAEs, systemic glucocorticoids may limit antitumor immunity and have their own substantial side effects, making long-term use risky [1, 6, 17, 18]. Developing treatment strategies that can reduce or replace systemic glucocorticoids while allowing patients to remain on immunotherapy is thus of substantial clinical importance [1, 6, 16].

The importance of endoscopic evaluation in the diagnosis of CPI enterocolitis is unclear [6–9, 19]. Current treatment guidelines recommend consideration of endoscopic evaluation in patients with severe symptoms [7–9]. Early endoscopy was associated with faster resolution of enterocolitis symptoms and shorter duration of glucocorticoid use in a retrospective analysis [20]. Endoscopy may be useful in identifying rare patients with CPI associated gastrointestinal symptoms that are not related to mucosal inflammation [21, 22], as well as patients with colonic ulceration who are more likely to fail initial management with glucocorticoids [10, 11]. Although mucosal changes are common in patients with CPI enterocolitis, a subset of patients have normal-appearing mucosa on endoscopy and lymphocyte-predominant inflammation [6, 23]. This syndrome shares features with the spontaneous colonic inflammatory disease microscopic colitis, which is distinct from other forms of inflammatory bowel disease. Microscopic colitis frequently responds to colonic formulations of budesonide, a glucocorticoid with high first-pass metabolism and low systemic absorption [24, 25].

In patients with enterocolitis from ipilimumab, prophylactic budesonide was found to be ineffective, although whether these results extend to therapeutic budesonide, or the subset of patients with only microscopic evidence of colitis, is unknown [26]. We performed a retrospective analysis of cases of CPI colitis without visible endoscopic inflammation at the Massachusetts General Hospital, a subset of which were treated with first-line budesonide, in order to describe clinical outcomes in this cohort.

## Methods

### Ethics

This retrospective analysis was approved by the Partners Human Research Committee, the Institutional Review Board of the Massachusetts General Hospital (MGH).

### Patients

We identified all patients ≥18 years of age who had prior CPI exposure and underwent standard-of-care flexible sigmoidoscopy from 3/1/2017 to 3/1/2019 for evaluation of suspected CPI enterocolitis.

### Definition of CPI microscopic colitis

**Fig. 1:**
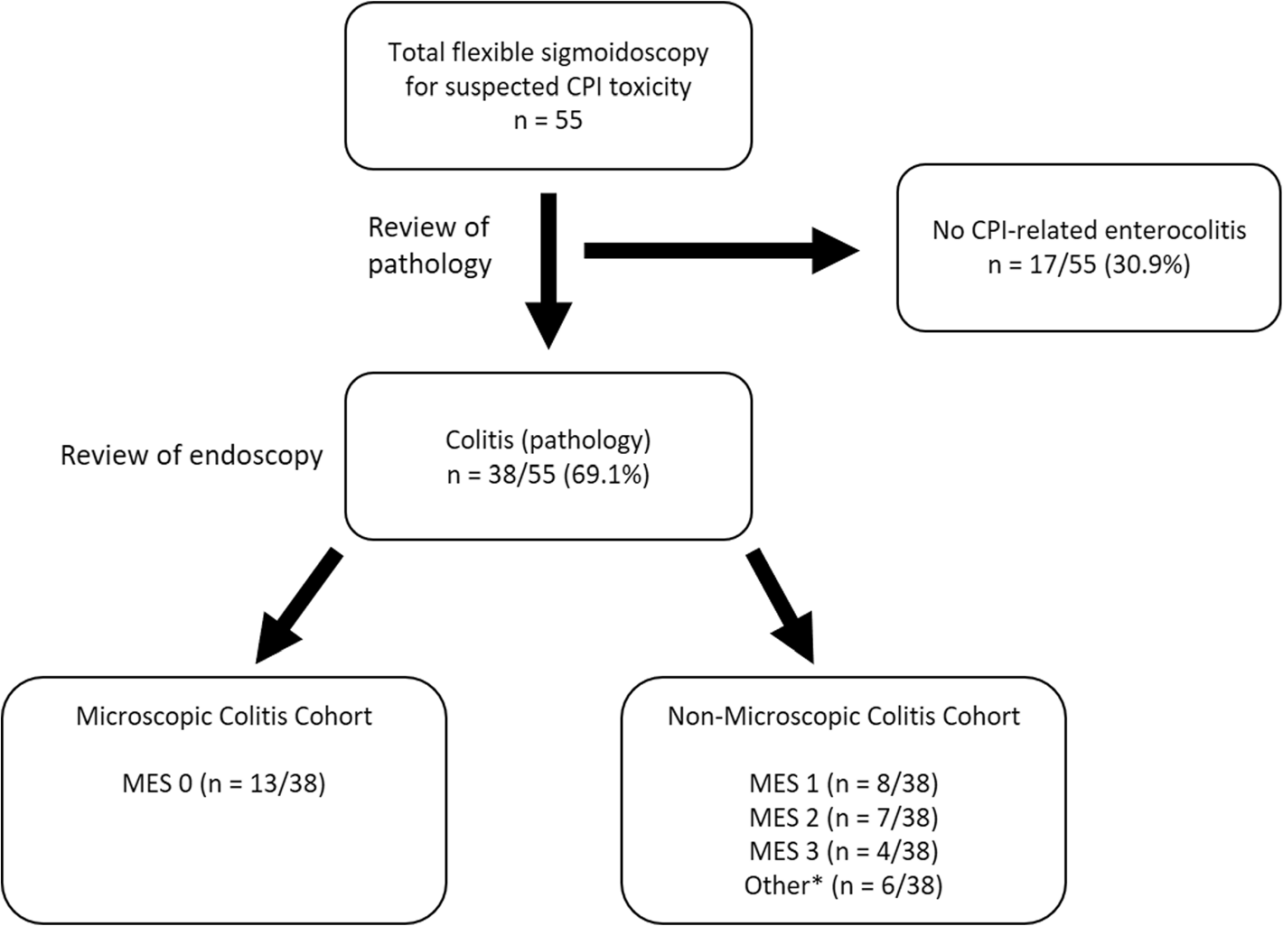
Cohort Selection. Patients were identified from all patients exposed to a CPI who underwent endoscopic evaluation for suspected CPI enterocolitis. *6 patients demonstrated upper GI tract inflammation in the absence of endoscopically visible colitis

CPI microscopic colitis was defined as clinical and histopathologic evidence of colitis without endoscopic inflammation (Mayo Endoscopic Score of 0) and without evidence of involvement of the upper gastrointestinal tract. Diagnoses were confirmed by two reviewers, one with clinical expertise in CPI complications (Fig. 1).

The Mayo Endoscopic Score (MES) is part of a clinical system devised at the Mayo Clinic, Rochester, Minnesota, that is used to quantify the degree of inflammation in the gastrointestinal tract for patients with ulcerative colitis [27]. The score ranges from 0 to 3: 0 indicates no features of macroscopic inflammation; 1 indicates mild inflammation, characterized by mild friability, erythema, and decreased vascularity; 2 indicates moderate inflammation, characterized by friability, marked erythema, absent vascular patterns, and presence of erosions; and 3 indicates severe inflammation, in which ulcerations and spontaneous bleeding are present.

### Data collection

We extracted clinical, laboratory, radiographic, and endoscopic data from electronic medical record (Additional file 1: Table S1). The National Cancer Institute’s Common Terminology Criteria for Adverse Events (CTCAE), version 4.0, was used for adverse event classification.

### Endpoints

Primary endpoints were time from symptom onset to resolution; absence of symptoms at 3 months after initial resolution, and discontinuation of CPI due to toxicity. Secondary endpoints were description of rate of admission for enterocolitis symptoms; incidence of new irAE development; and oncologic outcomes including time to treatment failure (TTTF), PFS, and OS.

### Histology

The colonic biopsies were reviewed by a gastrointestinal pathologist. The following patterns were scored in a blinded fashion: 1) Lymphocytic colitis-pattern, characterized by increased intraepithelial lymphocytes with or without cryptitis or crypt abscesses, 2) collagenous colitis-pattern characterized by thickened subepithelial collagen layer, and 3) acute self-limited pattern colitis characterized by intact crypt architecture with cryptitis and/or crypt abscesses.

### Statistical analysis

Patients were grouped in two primary ways for analysis: by whether or not they had microscopic colitis; and by whether or not they had received budesonide. Descriptive statistics were displayed using Microsoft Excel 2016 (Microsoft Corporation, Redmond, Washington, USA). Statistical analysis was performed using SAS Studio (version 9.4 M6, SAS Institute, Cary, NC, USA). Data are expressed as “mean +/- standard deviation,” “mean +/- standard error,” or “median (range)” where appropriate. *P*-values are two-sided, with α = 0.05.

The chi-square test or Fisher’s exact test and the ANOVA method or the Student’s *t*-test were employed where appropriate. Survival curves were generated using Kaplan-Meier analysis. Log-rank and Wilcoxon testing are reported where appropriate. Survival was measured from CPI exposure date to date of death, date of transition to hospice, or censored date. Date of death or transition to hospice was determined by electronic medical record review. Date of oncologic progression was defined as the date imaging was performed showing progressive disease.

## Results

### Characteristics and clinical course

**Fig. 2:**
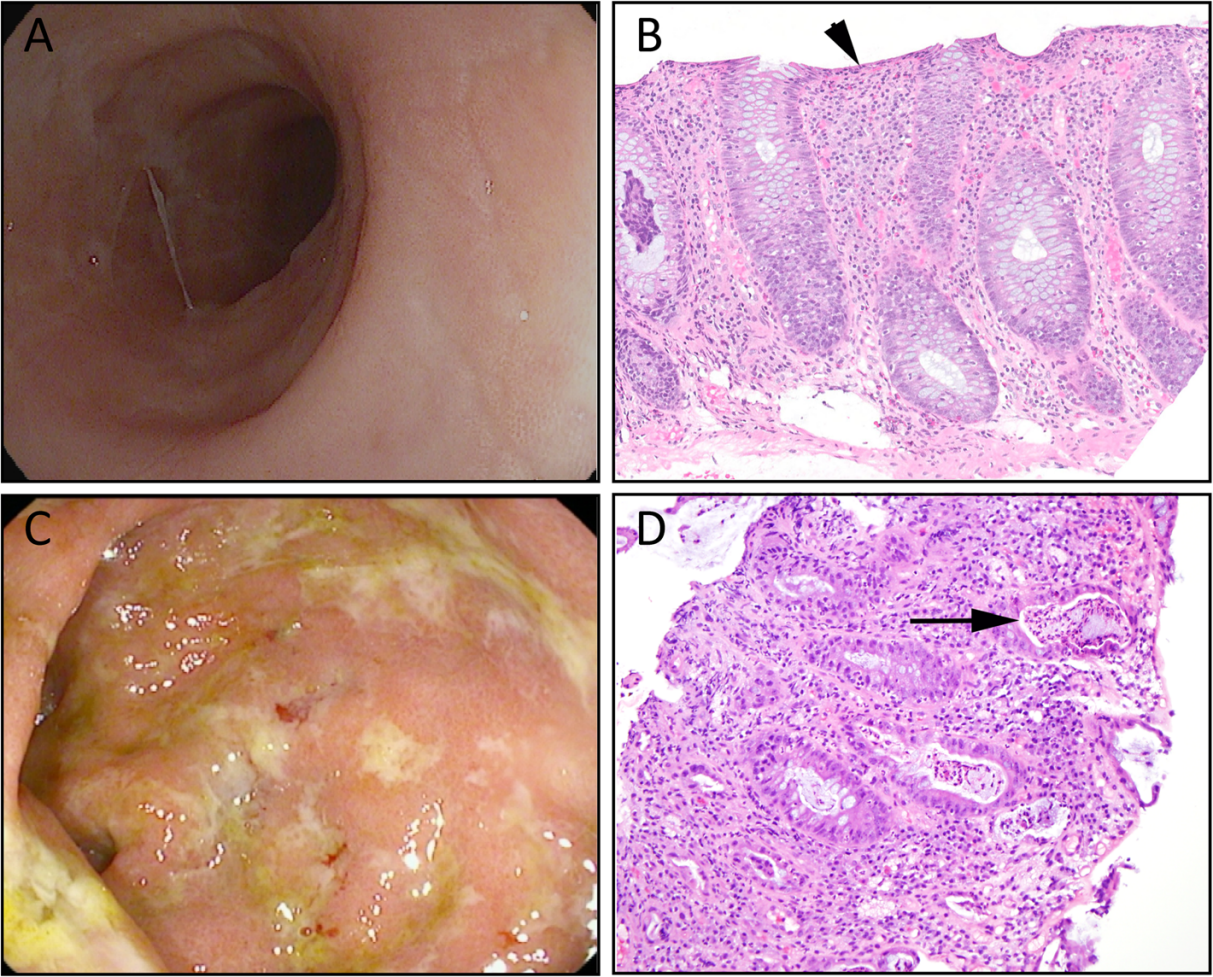
Endoscopic and histologic appearance of CPI microscopic colitis. **a** endoscopic image and H&E slide (**b**) from a patient with Mayo Endoscopic Score 0 microscopic colitis arising while on adjuvant nivolumab for stage III melanoma; (**b**) Lymphocytic-pattern colitis. Note the intact crypt architecture and increased intraepithelial lymphocytes (arrow). (**c**) endoscopic image and H&E slide (**d**) from a patient with Mayo Endoscopic Score 3 colitis arising while on adjuvant nivolumab for stage III melanoma; (**d**) Acute self-limiting pattern injury. Note the intact crypt architecture and crypt abscesses (arrow)

From 2017 to 2019, 55 patients were evaluated by endoscopy for suspected CPI enterocolitis (Fig. 1). 38 patients with CPI enterocolitis were identified by endoscopy from 3/01/2017 to 3/01/2019 out of the 55 who underwent endoscopy. 13/38 (34.2%) patients had biopsy-confirmed colitis in the absence of enteritis with a Mayo Endoscopic Score (MES) of 0 (microscopic colitis) (Fig. 2). Nineteen patients had enterocolitis with an MES of at least 1 indicating macroscopic inflammation (Fig. 2); in addition, five patients had pathologic evidence of enteritis and colitis without endoscopic evidence of mucosal inflammation, and one patient had an MES that could not be determined due to stool that interfered with mucosal visualization. Together these 25/38 patients composed the non-microscopic colitis cohort.

### Histology

Histologically, patients with MES of 0 showed either lymphocytic colitis-pattern injury (12/13) or collagenous colitis-pattern injury (1/13); 6 of the 12 cases with lymphocytic-pattern injury also showed foci of cryptitis. All 12 patients with MES of > = 1 whose slides were available for review showed an acute self-limiting colitis-pattern of injury.

### Case series

In-depth review of the medical record was performed for the patients evaluated between 3/1/2017 and 3/1/2019 who were found to have CPI enterocolitis. Baseline characteristics are summarized in Table 1, Additional file 1: Tables S2 and S3 with univariate analyses stratified by enterocolitis subset. Of the 38 patients with confirmed CPI enterocolitis, melanoma and non small cell lung cancer (NSCLC) were the most common underlying malignancies, though multiple advanced-stage hematologic and solid malignancies were represented (Table 1). Prior irAEs were uncommon (Additional file 1: Table S2). Metastases to the gastrointestinal mucosa were uncommon in both cohorts (Additional file 1: Table S3). A total of 20/38 (52.6%) had macroscopically visible CPI enterocolitis, with the distribution approximately evenly split among MES 1–3 (Fig. 1). Average age at endoscopy for the microscopic colitis cohort was 62 years and 7/13 (53.8%) were male, which did not differ statistically from the non-microscopic colitis cohort (Table 1). Patients in both cohorts were treated primarily with PD-1/PD-L1 therapies (microscopic colitis: 11/13, 84.6%; non-microscopic colitis: 16/25, 64.0%) (Table 1). Average CTCAE grade was 2 for both cohorts and its distribution did not show a significant difference between the groups (*p* = 1.000) (Table 1). Initial chemistries and blood counts were typically within or near the normal ranges. Slight lymphopenia with corresponding neutrophilia was noted in both cohorts (Additional file 1: Table S3).

**Table 1:** Baseline characteristics

	Overall	Microscopic colitis	Non-microscopic colitis	*p*-value
Number of patients	38	13	25	1.000
Age in years				
Mean +/− SD	62.3 +/− 8.9	62.4 +/− 8.6	62.2 +/− 9.2	0.952
Median	62.5	62.0	64.0	
Sex (M:F)	17:21	7:6	14:11	0.899
CPI regimen				
α-CTLA-4	3/38 (7.9%)	1/13 (7.7%)	2/25 (8.0%)	0.281
α-PD-(L)1	27/38 (71.1%)	11/13 (84.6%)	16/25 (64.0%)	
Combination CPI	8/38 (21.1%)	1/13 (7.7%)	7/25 (28.0%)	
Tumor type				
Melanoma	21/38 (55.3%)	6/13 (46.2%)	15/25 (60.0%)	0.415
NSCLC	7/38 (18.4%)	3/13 (23.1%)	4/25 (16.0%)	0.672
Other	10/38 (26.3%)	4/13 (30.8%)	6/25 (24.0%)	0.709
Prior irAE	14/38 (36.8%)	5/13 (38.5%)	11/25 (44.0%)	0.743
Prior medications				
Antibiotics within 3 months	9/38 (23.7%)	2/13 (15.4%)	7/25 (28.0%)	0.456
PPI	14/38 (36.8%)	7/13 (53.9%)	7/25 (28.0%)	0.163
SSRI/SNRI	10/38 (26.3%)	4/13 (30.8%)	6/25 (24.0%)	0.709
Estrogen	3/35 (7.9%)	2/13 (15.4%)	1/25 (4.0%)	0.265
Time from first CPI infusion to symptom onset (days)				
Mean +/− SD	139.5 +/− 153.9	225.0 +/− 214.9	95.1 +/− 86.1	0.011*
Median	72.5	150.0	68.0	
CTCAE symptom grade: median (IQR)	2 (1–3)	2 (1–3)	2 (1–3)	1.000

The p-value was calculated by ANOVA for numerical covariates and chi-square test or Fisher’s exact for categorical covariates, where appropriate. Combination CPI: all patients received a PD-(L)1 inhibitor in combination with ipilimumab either as standard of care or on an investigational protocol. SD: standard deviation. CPI: immune checkpoint inhibitor. PD-(L)1: programmed cell death receptor (ligand)-1. NSCLC: non-small cell lung cancer. Other tumor types: breast cancer (n = 1); colorectal cancer (*n* = 1); cutaneous squamous cell cancer (*n* = 1); squamous cell cancer of the head and neck (*n* = 1); diffuse large B-cell lymphoma (*n* = 2); multiple myeloma (*n* = 1); ovarian adenocarcinoma (*n* = 2); renal cell cancer (*n* = 1). PPI: proton-pump inhibitor. SSRI/SNRI: selective serotonin reuptake inhibitor/serotonin and norepinephrine reuptake inhibitor. Prior medications were within one year of symptoms unless otherwise specified. CTCAE: Common Terminology Criteria for Adverse Events. IQR: interquartile range. *statistically significant at significance of 0.05

All of the boldfaced numbers should be statistically signficant

Proton-pump inhibitors (PPIs), selective serotonin reuptake inhibitors (SSRIs), and estrogen hormonal therapy have been associated with an increased risk of spontaneous microscopic colitis in epidemiologic studies [24, 25]. We found no association with recent exposure to PPIs (7/13, 53.9%), SSRIs (4/13, 30.8%), or estrogen (2/13, 15.4%) and the development of microscopic colitis compared to non-microscopic colitis, though for each of these drug classes the frequency of use was numerically higher in the microscopic colitis cohort.

We assumed that the patient’s most recent immunotherapeutic regimen was responsible for the development of microscopic colitis, and we defined the patient’s initial CPI exposure by the first infusion of this treatment regimen. Time to symptom onset from initial CPI exposure occurred a median of 150 days after initiation of CPIs in the microscopic colitis cohort compared to 68 days in the non-microscopic colitis cohort (Table 1, *p* = 0.011). Time from symptom onset to medical evaluation did not differ significantly between the two groups. Common presenting symptoms included diarrhea (microscopic colitis: 13/13, 100.0%; non-microscopic colitis: 24/25, 96.0%). Abdominal pain and urgency were less common and did not differ between the two cohorts (Additional file 1: Table S3).

### Primary endpoint assessment

**Fig. 3:**
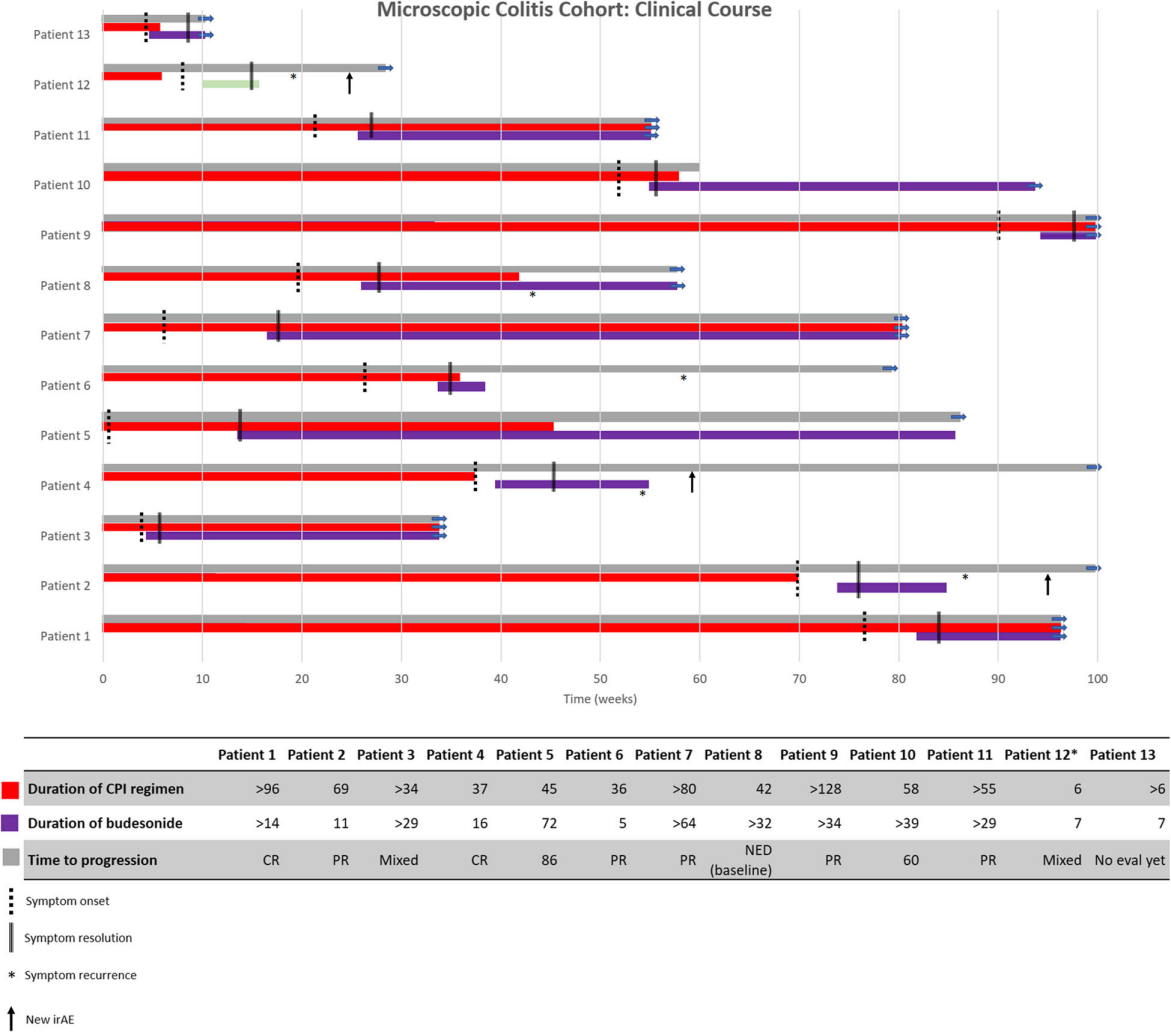
CPI microscopic colitis clinical course. Summary of immunotherapy treatment history, symptom onset and duration, and initiation of budesonide for the 13 patients in the microscopic colitis cohort. Patient 12 received systemic guideline-dose glucocorticoids, represented in green below

The overall clinical course of each patient with microscopic colitis is summarized in Fig. 3. Median time from first CPI exposure to first glucocorticoid use was 258.2 days for the microscopic colitis cohort but 120.6 days for the non-microscopic colitis cohort (*p* = 0.010), consistent with the later onset of symptoms in patients with microscopic colitis (Additional file 1: Table S3). 12/13 (92.3%) patients with microscopic colitis were treated with budesonide, compared to 3/25 (12.0%) patients with non-microscopic CPI enterocolitis who were treated with budesonide in addition to other glucocorticoids. Systemic glucocorticoid use was significantly more common in the non-microscopic colitis cohort (22/25, 88.0%) than in the microscopic colitis cohort (3/13, 23.1%, *p* < 0.001), though glucocorticoids were initiated within similar periods of time for each cohort (Table 2). Median time from symptom onset to resolution did not differ between cohorts (microscopic colitis: 50.1 days; non-microscopic colitis: 49.9 days; *p* = 0.985) (Additional file 1: Table S3). More than 80% of microscopic colitis and non-microscopic colitis patients were GI symptom-free at 3 months after initial resolution (*p* = 1.000) (Additional file 1: Table S3).

**Table 2:** Colitis outcomes

	Overall	Microscopic colitis	Non-microscopic colitis	*p*-value
Interventions				
Budesonide	15/38 (39.5%)	12/13 (92.3%)	3/25 (12.0%)	< 0.001*
Any systemic glucocorticoids	25/38 (65.8%)	3/13 (23.1%)	22/25 (88.0%)	< 0.001*
Systemic glucocorticoids < 1 mg/kg/d	14/38 (36.8%)	2/13 (15.4%)	12/25 (48.0%)	0.077
Systemic glucocorticoids ≥1 mg/kg/d	12/38 (31.2%)	1/13 (7.7%)	11/25 (44.0%)	0.030*
Time from symptom onset to initiation of glucocorticoids (days)				
Mean +/− SD	32.4 +/− 37.1	33.2 +/− 26.0	32.0 +/− 42.7	0.928
Median	21.0	28.0	18.0	
Proportion continuing CPI course	14/38 (36.8%)	10/13 (76.9%)	4/25 (16.0%)	< 0.001*
Proportion eventually discontinuing immunotherapy	29/38 (76.3%)	8/13 (61.5%)	21/25 (84.0%)	0.226
Due to toxicity alone	22/38 (57.9%)	6/13 (46.2%)	16/25 (64.0%)	1.000
Due to any progressive disease	7/38 (18.4%)	2/13 (15.4%)	5/25 (25.0%)	
Average number of additional infusions^#^	3.0 +/− 5.7	5.8 +/− 6.8	1.6 +/− 4.5	0.030*
Additional irAEs after colitis	8/38 (21.1%)	3/13 (23.1%)	5/25 (20.0%)	1.000
Response to first line treatment	22/38 (67.9%)	9/13 (69.2%)	14/25 (56.0%)	0.429
Proportion of patients requiring second-line immunosuppression	23/38 (60.5%)	5/13 (38.5%)	18/25 (72.0%)	Not calculated
Proportion requiring TNFα inhibitor	16/38 (42.1%)	4/13 (30.8%)	11/25 (44.0%)	0.429

The *p*-value was calculated by ANOVA for numerical covariates and chi-square test or Fisher’s exact for categorical covariates, where appropriate. TNFα: tumor necrosis factor α. CPI: immune checkpoint inhibitor. SD: standard deviation. irAE: immune-related adverse event. ^#^calculated among patients who continued CPIs. *denotes statistical significance at *p* = 0.05. Second-line immune suppression includes exposure to different glucocorticoids in the microscopic colitis cohort

All of the boldfaced numbers should be statistically signficant

Patients in the microscopic colitis cohort were significantly more likely to remain on their CPI than were patients with non-microscopic colitis (76.9% versus 16.0%, *p* < 0.001) (Table 2). For patients who remained on their CPI, patients with microscopic colitis received more additional treatment than did patients with non-microscopic colitis (average of 5.8 versus 1.6 additional infusions, *p* = 0.03) (Table 2).

### Secondary endpoint assessment

Less than half of each cohort was admitted for enterocolitis symptoms; although patients with non-microscopic colitis were admitted more often, this did not reach statistical significance (microscopic colitis: 2/13, 15.4%; non-microscopic colitis: 11/25, 45.0%; *p* = 0.148). For patients who were admitted, median length of stay was approximately one week in both cohorts. Patients in both cohorts developed additional irAEs after development of colitis (microscopic colitis: 3/13, 23.1%; non-microscopic colitis 11/25, 44.0%) (Table 2). Secondary immune suppression for (entero)colitis, including TNFα inhibitors, were used at similar rates in both cohorts (Table 2).

We characterized oncologic outcomes associated with CPI microscopic colitis (Additional file 1: Figure S1). Median follow-up time was 18.8 months. The timeframe of our study precluded the presentation of meaningful OS data, and our small sample size precluded analysis stratified by tumor type. Univariate Cox regression for the effect of microscopic colitis on TTTF showed a hazard ratio of 0.30 (95% CI 0.14–0.66); similar Cox regression analysis for effect on PFS showed a hazard ratio of 0.22 (95% CI 0.07–0.70).

## Discussion

We defined a subset of CPI associated colitis that we term “CPI microscopic colitis” that can be identified endoscopically and responds to colonic formulated budesonide, enabling treatment of this toxicity without the use of systemic glucocorticoids and while continuing immunotherapy for the underlying malignancy. The definition of CPI microscopic colitis that we use is based on mucosal assessment by endoscopy paired with biopsies; these patients have no mucosal evidence of inflammation (Mayo Endoscopic Score 0) but have lymphocytic/collagenous-pattern colitis on histopathology. We excluded patients with concurrent enteritis from this definition, as small intestinal inflammation is difficult to treat with currently available budesonide formulations, and thus such patients behave differently in the setting of available treatments. Whether this cohort definition identifies a distinct pathologic entity, or a milder subtype of CPI enterocolitis with a distinct treatment response, is unclear [6, 23]. In this retrospective analysis, we offer an estimate of CPI microscopic colitis incidence with approximately a third of our patients with mucosal inflammation falling into this group. We further describe key features of the typical disease course, and compare them to non-microscopic colitis. In our cohort, budesonide was effective as first-line treatment for CPI microscopic colitis, as it is in patients who develop spontaneous microscopic colitis [24, 25]. Importantly, many of our patients were able to remain on immunotherapy after initiation of budesonide. Although immunotherapy was ultimately discontinued in most patients, often for the development of another irAE, several patients in the cohort were able to complete their immunotherapy treatment course while on budesonide.

Most of the patients in our cohort were identified by flexible sigmoidoscopy paired with a negative upper endoscopy. Although CPI colitis can often have regional variability, approximately 95% of patients have disease on the left side, which would be observable by flexible sigmoidoscopy [28]. For most of our cohort, we cannot exclude the possibility that right-sided mucosal injury would have been apparent had a full colonoscopy been performed; however, our data suggest that colitis occurring in the absence of left-sided mucosal injury can be treated with budesonide, regardless of whether information about the right colon is available. Determining whether isolated right-sided colitis is a rare cause of failure to respond to budesonide in otherwise apparent CPI microscopic colitis will require evaluation of larger cohorts. Upper gastrointestinal inflammation occurred in 39.5% (15/38) of our cohort, either in isolation or paired with colitis, indicating that gastric and duodenal inflammation is common in patients with gastrointestinal toxicities from CPIs and may be an important cause of diarrhea in patients on CPIs who do not have colitis on lower endoscopy [29].

From the range of cancers represented in our cohort, we suggest that CPI microscopic colitis occurs across cancer types, indicating a relationship to the immunotherapeutic agent rather than to cancer-specific factors. The relatively large proportion of melanoma and non-small cell lung cancer in our cohorts likely reflects the prevalence of those cancers among patients on CPIs more generally. We did not find a female preponderance in our study, and in our analysis of the prevalence of selected known risk factors for spontaneous microscopic colitis, we found no predictors of disease [24]. Proton pump inhibitor use and hormonal exposure in particular were more common in the microscopic colitis cohort, though this finding did not reach statistical significance. Larger analyses will be necessary in order to definitively determine whether such an association exists, as has been reported for spontaneous microscopic colitis [24, 25].

We identified few clinical distinctions between CPI microscopic colitis and enterocolitis presenting with mucosal signs of inflammation, aside from the endoscopic features used to define these cohorts. CPI microscopic colitis and non-microscopic colitis were indistinguishable by CTCAE grade on presentation, as well as by routine laboratory testing. The frequency of microscopic colitis was numerically higher in patients treated with single agent PD-(L)1 blockade, though this association did not reach statistical significance in this cohort. Nevertheless, the finding is suggestive that combination immunotherapy, which induces more frequent colitis, may also lead to more significant mucosal injury. We did find that the time interval between CPI exposure and symptom onset was longer for CPI microscopic colitis (median 150.0 days) than for non-microscopic colitis (median 68.0 days), though the intervals between the two cohorts overlapped enough to preclude an accurate diagnosis of microscopic colitis using time of onset alone. The absence of other clear indicators of CPI microscopic colitis, and the availability of a specific management strategy (i.e. local glucocorticoids) underscores the potential value of early endoscopic evaluation in patients with suspected CPI enterocolitis. This subset of CPI enterocolitis appears to be common (approximately 1/3 of our total CPI enterocolitis cohort), and the use of budesonide for treatment could not only prevent the use of systemic glucocorticoids, but also enable some patients to received further immunotherapy.

Most patients who develop enterocolitis from CPIs, regardless of the severity of mucosal inflammation, will eventually discontinue CPI treatment due to toxicity; these findings are in line with prior literature [10, 11, 15]. Absence of recrudescence after initial symptom control was achieved in over 80% of the microscopic colitis cohort. As the patients with microscopic colitis generally continued to receive immunotherapy for longer than those patients with severe CPI enterocolitis, we would expect a higher incidence of dose or time dependent adverse events. The incidence of novel irAE development in our cohort (23.7%) is consistent with prior studies on overall CPI enterocolitis rechallenged with immunotherapy, and was statistically identical between the two groups [30].

Our survival analyses of TTTF and PFS are intriguing, though confounded by multiple variables in our heterogeneous cohort, including different underlying stage and type of malignancy, the specific therapies used, and duration of therapy prior to onset of toxicity. Budesonide use was statistically significantly associated with decreased risk of treatment failure (HR 0.28). The heterogeneous mixture of treatments and malignancies in our cohort coupled with its small size reduce our ability to determine the clinical importance of this finding. Although consistent with a beneficial effect of local glucocorticoid delivery on antitumor immunity, the reduction in the risk of treatment failure could also reflect the longer duration of CPI use prior to symptom onset, or, less likely, differences in underlying biology between microscopic and non-microscopic colitis induced by CPIs. Ultimately, prospective analyses with more uniform cohorts will be necessary to determine whether these preliminary cancer outcome findings are clinically meaningful.

Our study’s retrospective nature precluded causal inference and introduced inherent survival bias, and our small sample size precluded multivariate regression; we performed univariate Cox regression modeling to ensure that we did not overfit our data. The relatively short time frame of the study precluded a long-term survival analysis. Several of our variables were highly correlated, limiting our ability to parse out their individual effects and introducing potential codependence into our findings. Most patients in the cohort received PD-1 or PD-L1 inhibitors, reflecting current practice but also potentially reflecting differences in the risk for this syndrome according to immunotherapeutic agent. Our sample size also precluded stratification by tumor type or stage.

## Conclusion

CPI microscopic colitis is a common subset of CPI enterocolitis that is distinct from both spontaneous microscopic colitis and other forms of CPI enterocolitis. Currently, endoscopy is the only method for distinguishing CPI microscopic colitis from other forms of CPI associated mucosal inflammation. In contrast to unselected CPI enterocolitis, budesonide appears to be an effective first-line treatment for CPI microscopic colitis and prolongs time on immunotherapy, while reducing exposure to systemic glucocorticoids. These findings provide a compelling rationale for the routine use of endoscopy in the stratification of patients with suspected gastrointestinal inflammation on CPIs, and suggest a reasonable alternative treatment strategy for patients with CPI-induced mucosal inflammation but without visible evidence of mucosal injury.

## Supplementary information

**Additional file 1: Table S1.** Full listing of data collection variables determined *a priori* from clinical experience, sorted alphabetically. **Table S2.** Additional characteristics of prior irAEs. Prior irAEs defined as symptom manifestations of any adverse reaction felt related to CPI therapy, before onset of colitis. Any prior gastrointestinal irAEs that occurred while the patient was receiving a different CPI regimen. 3/38 (7.9%) patients had multiple prior irAEs. **Table S3.** Additional features and results characterizing patient presentations and clinical courses. Univariate analysis by colitis subset displayed. Enterocoltis symptoms were inquired after at standard oncologic follow-up visits. Of note, the total number of patients decreased over time, yielding decreasing denominators in “Absence of symptom recrudescence.” **Figure S1.** Kaplan-Meier survival curves for TTTF and PFS. ** denotes significance at α<0.05. (a) TTTF, stratified by colitis type. (b) PFS, stratified by colitis type. One patient’s clinical response to CPI therapy had not yet been evaluated at time of data collection.

## Data Availability

Not applicable
