# Supplementary material for: Budesonide treatment for microscopic colitis from immune checkpoint inhibitors
Source: J Immunother Cancer. 2019 Nov 7;7:292. doi: 10.1186/s40425-019-0756-0 (PMC6839080; doi:10.1186/s40425-019-0756-0)

**Supplemental Table 1**

Full listing of data collection variables determined *a priori* from clinical experience, sorted alphabetically.

| Additional comments |
| --- |
| Age of patient at time of admission |
| Antibiotics within one month of admission |
| Antibiotics within three months of admission |
| Any admission for GEC symptoms? |
| Budesonide administered vs. not administered |
| Budesonide administration: duration |
| Budesonide administration: end date |
| Budesonide administration: normalized peak dose |
| Budesonide administration: peak dose |
| Budesonide administration: reason for discontinuation |
| Budesonide administration: start date |
| Budesonide administration: was uptitration necessary? |
| CPI administration: reason for discontinuation |
| CTCAE grade at admission |
| Date of birth |
| Date of first CPI administration (most proximate to GEC) |
| Date of first medical evaluation at the Massachusetts General Hospital |
| Date of last CPI administration prior to symptoms |
| Date of symptom onset |
| Development of additional irAEs |
| Diagnosis: colitis, enterocolitis, gastroenterocolitis |
| ECOG performance status: at admission |
| ECOG performance status: at initial CPI administration |
| ECOG performance status: worst over clinical course |
| Endoscopy: Date performed, initial evaluation |
| Endoscopy: Findings |
| Endoscopy: Histopathologic findings |
| Endoscopy: Mayo Endoscopic Score, initial evaluation |
| Endoscopy: Modality |
| If admitted: how many admissions for GEC symptoms? |
| Immunosuppressive regimen: alternative immunosuppressive regimen |
| Immunosuppressive regimen: complications |
| Immunosuppressive regimen: exposure to high-dose steroids |
| Immunosuppressive regimen: exposure to low-dose steroids only |
| Immunosuppressive regimen: TNFαi administration |
| Infectious disease testing: assays and results, inpatient |
| Infectious disease testing: assays and results, outpatient |
| Inpatient immunosuppressive regimen |
| Laboratory testing: Absolute eosinophil count (upon initial medical evaluation after symptom onset) |
| Laboratory testing: Absolute lymphocyte count (upon initial medical evaluation after symptom onset) |
| Laboratory testing: Absolute neutrophil count (upon initial medical evaluation after symptom onset) |
| Laboratory testing: Albumin (upon initial medical evaluation after symptom onset) |
| Laboratory testing: Blood urea nitrogen (upon initial medical evaluation after symptom onset) |
| Laboratory testing: C-reactive protein (upon initial medical evaluation after symptom onset) |
| Laboratory testing: Erythrocyte sedimentation rate (upon initial medical evaluation after symptom onset) |
| Laboratory testing: Ferritin level (upon initial medical evaluation after symptom onset) |
| Laboratory testing: Hematocrit (upon initial medical evaluation after symptom onset) |
| Laboratory testing: Hemoglobin (upon initial medical evaluation after symptom onset) |
| Laboratory testing: Lactate dehydrogenase (upon initial medical evaluation after symptom onset) |
| Laboratory testing: Leukocyte count (upon initial medical evaluation after symptom onset) |
| Laboratory testing: Platelet count (upon initial medical evaluation after symptom onset) |
| Laboratory testing: Relative eosinophil count (upon initial medical evaluation after symptom onset) |
| Laboratory testing: Relative lymphocyte count (upon initial medical evaluation after symptom onset) |
| Laboratory testing: Relative neutrophil count (upon initial medical evaluation after symptom onset) |
| Laboratory testing: Serum chloride (upon initial medical evaluation after symptom onset) |
| Laboratory testing: Serum creatinine (upon initial medical evaluation after symptom onset) |
| Laboratory testing: Serum potassium (upon initial medical evaluation after symptom onset) |
| Laboratory testing: Serum sodium (upon initial medical evaluation after symptom onset) |
| Laboratory testing: Tissue transglutaminase IgA (upon initial medical evaluation after symptom onset) |
| Length of stay, if any |
| Non-glucocorticoid hormone administration within one year of admission |
| Number of additional doses of CPI after symptom onset |
| Number of cycles prior to colitis |
| Oncologic outcomes at time of symptom onset |
| Oncologic outcomes: clinical course after colitis |
| Oncologic outcomes: most recent response to CPI to date |
| Oncologic outcomes: overall survival |
| Oncologic outcomes: progression-free survival |
| Oncologic outcomes: time to treatment failure |
| Outcomes, endoscopy: date of follow-up evaluation |
| Outcomes, endoscopy: findings at follow-up evaluation |
| Outcomes, endoscopy: histopathologic findings at follow-up evaluation |
| Outcomes, endoscopy: modality of follow-up evaluation |
| Outcomes, radiography: date of follow-up evaluation |
| Outcomes, radiography: findings at follow-up evaluation |
| Outcomes: date of first resolution to grade 1 symptoms or better |
| Outcomes: interventions and course at one month from symptom resolution |
| Outcomes: interventions and course at six months from symptom resolution |
| Outcomes: interventions and course at three months from symptom resolution |
| Outcomes: interventions and course at twelve months from symptom resolution |
| Outcomes: interventions and course at two months from symptom resolution |
| Outcomes: recrudescence at one month from symptom resolution |
| Outcomes: recrudescence at six months from symptom resolution |
| Outcomes: recrudescence at three months from symptom resolution |
| Outcomes: recrudescence at twelve months from symptom resolution |
| Outcomes: recrudescence at two months from symptom resolution |
| Outcomes: time to first resolution from immunosuppressive regimen initiation |
| Outcomes: time to first resolution from symptom onset |
| Presence of abdominal visceral metastases |
| Presence of gastrointestinal metastases |
| Presence of hepatic metastases |
| Prior history of autoimmune disease |
| Prior history of inflammatory bowel disease |
| Prior irAEs |
| Prior treatments |
| Proton pump inhibitor use within one year of admission |
| Radiographic testing |
| Recorded electronic medical record ethnicity |
| Regimen of checkpoint inhibition received |
| Serotonin modulator use within one year of admission |
| Sex of patient |
| Stage of cancer |
| Study identifier |
| Symptoms/signs at presentation |
| Time to first medical contact from symptom onset |
| Time to first medical evaluation from initial CPI administration |
| Time to first medical evaluation from most recent CPI administration |
| Time to symptom onset from initial CPI administration |
| Time to symptom onset from most recent CPI administration |
| Treatment: Date of first high-dose steroid exposure |
| Treatment: date of first steroid exposure |
| Treatment: date of steroid discontinuation |
| Treatment: symptom duration until any treatment |
| Treatment: systemic steroid administration prior to budesonide |
| Treatment: time from first CPI administration to first steroid exposure |
| Treatment: time from initial CPI administration to first high-dose steroid exposure |
| Treatment: Time from symptom onset to first high-dose steroid exposure |
| Treatment: time from symptom onset to first steroid exposure |
| Treatment: were non-immunosuppressive medications used for symptom control? |
| Tumor: established genetic markers |
| Tumor: microsatellite status |
| Tumor: PD-1 expression level |
| Type of cancer |
| Weight at budesonide administration |

**Supplemental Table 2**

Additional characteristics of prior irAEs. Prior irAEs defined as symptom manifestations of any adverse reaction felt related to CPI therapy, before onset of colitis. Any prior gastrointestinal irAEs occurred while the patient was receiving a different CPI regimen. 3/38 (7.9%) patients had multiple prior irAEs.

| **Table S2: Features of prior irAEs** | | | | | | |
| --- | --- | --- | --- | --- | --- | --- |
|  | | Overall | Microscopic colitis | | Non-microscopic colitis | |
| Ocular | | | | | | |
|  | Episcleritis | 1/38 (2.6%) | | 0/13 (0.0%) | | 1/25 (4.0%) |
| Pulmonary | | | | | | |
|  | Pneumonitis | 2/38 (5.3%) | | 0/13 (0.0%) | | 2/25 (8.0%) |
| Gastrointestinal | | | | | | |
|  | Hepatitis | 2/38 (5.3%) | | 1/13 (7.7%) | | 1/25 (4.0%) |
|  | Gastritis | 1/38 (2.6%) | | 0/13 (0.0%) | | 1/25 (4.0%) |
|  | Enterocolitis | 2/38 (5.3%) | | 1/13 (7.7%) | | 1/25 (4.0%) |
| Endocrinologic | | | | | | |
|  | Thyrotoxicosis | 1/38 (2.6%) | | 0/13 (0.0%) | | 1/25 (4.0%) |
|  | Adrenalitis | 1/38 (2.6%) | | 0/13 (0.0%) | | 1/25 (4.0%) |
|  | Hypophysitis | 1/38 (2.6%) | | 0/13 (0.0%) | | 1/25 (4.0%) |
| Hematologic | | | | | | |
|  | Pancytopenia | 1/38 (2.6%) | | 0/13 (0.0%) | | 1/25 (4.0%) |
|  | Hemophagocytic lymphohistiocytosis | 1/38 (2.6%) | | 0/13 (0.0%) | | 1/25 (4.0%) |
| Rheumatologic | | | | | | |
|  | Inflammatory arthritis | 3/38 (7.9%) | | 1/13 (7.7%) | | 2/25 (8.0%) |
|  | Sarcoidotic characteristics on imaging, asymptomatic | 1/38 (2.6%) | | 1/13 (7.7%) | | 0/25 (0.0%) |

**Supplemental Table 3**

Additional features and results characterizing patient presentations and clinical courses. Univariate analysis by colitis subset displayed. GEC symptoms were inquired after at standard oncologic follow-up visits. Of note, the total number of patients decreased over time, yielding decreasing denominators in “Absence of symptom recrudescence.”

| **Table S3: Additional features of clinical course** | | | | | | | | | | | | | | |
| --- | --- | --- | --- | --- | --- | --- | --- | --- | --- | --- | --- | --- | --- | --- |
|  | | | | Overall | | | Microscopic colitis | | | Non-microscopic colitis | | p-value | | |
| Time from most recent CPI infusion to symptom onset (days) | | | | | | | | | | | | | | |
|  | Mean +/- SD | | | 12.9 +/- 15.4 | | | 7.2 +/- 6.6 | | | 15.9 +/- 17.8 | | 0.100 | | |
|  | Median | | | 8.0 | | | 5.0 | | | 10.0 | |  |  |  |
| Time from symptom onset to first medical contact (days) | | | | | | | | | | | | | | |
|  | | Mean +/- SD | | 13.6 +/- 31.0 | | | 6.6 +/- 5.9 | | | 17.3 +/- 37.7 | | 0.321 | | |
|  | | Median | | 4.5 | | | 5.0 | | | 4.0 | |  |  |  |
| Presenting signs and symptoms | | | | | | | | | | | | | | |
|  | | Diarrhea | | 37/38 (97.4%) | | | 13/13 (100.0%) | | | 24/25 (96.0%) | | 1.000 | | |
|  | | Nausea and/or vomiting | | 6/38 (15.8%) | | | 2/13 (15.4%) | | | 4/25 (16.0%) | | 1.000 | | |
|  | | Abdominal pain | | 16/38 (42.1%) | | | 5/13 (38.5%) | | | 11/25 (44.0%) | | 0.743 | | |
|  | | Urgency | | 14/38 (36.8%) | | | 6/13 (46.2%) | | | 8/25 (32.0%) | | 0.486 | | |
|  | | Fecal incontinence | | 6/38 (15.8%) | | | 2/13 (15.4%) | | | 4/25 (16.0%) | | 1.000 | | |
|  | | Melena and/or hematochezia | | 1/38 (2.6%) | | | 0/13 (0.0%) | | | 1/25 (4.0%) | | 1.000 | | |
|  | | Bloating | | 4/38 (10.5%) | | | 1/13 (7.7%) | | | 3/25 (12.0%) | | 1.000 | | |
|  | | Weight loss | | 3/38 (7.9%) | | | 1/13 (7.7%) | | | 2/25 (8.0%) | | 1.000 | | |
|  | | Epigastric burning | | 1/38 (2.6%) | | | 0/13 (0.0%) | | | 1/25 (4.0%) | | 1.000 | | |
| Laboratory results at symptom evaluation | | | | | | | | | | | | | | |
|  | Serum sodium (mmol/L) | | | 138 +/- 4 | | | 140 +/- 2 | | | 138 +/- 5 | | 0.111 | | |
|  | Serum potassium (mmol/L) | | | 4.1 +/- 0.5 | | | 4.3 +/- 0.4 | | | 4.1 +/- 0.5 | | 0.197 | | |
|  | Serum chloride (mmol/L) | | | 101 +/- 4 | | | 102 +/- 2 | | | 100 +/- 4 | | 0.134 | | |
|  | Blood urea nitrogen (mg/dL) | | | 16 +/- 8 | | | 16 +/- 7 | | | 16 +/- 8 | | 0.928 | | |
|  | Serum creatinine (mg/dL) | | | 0.9 +/- 0.3 | | | 1.0 +/- 0.3 | | | 0.9 +/- 0.3 | | 0.863 | | |
|  | Leukocytes (K cells/mL) | | | 8.06 +/- 5.62 | | | 6.95 +/- 1.80 | | | 8.63 +/- 6.78 | | 0.389 | | |
|  | Hgb (g/dL) | | | 13.1 +/- 2.1 | | | 13.5 +/- 1.8 | | | 12.9 +/- 2.3 | | 0.474 | | |
|  | Hct (%) | | | 39.4 +/- 5.6 | | | 40.1 +/- 4.1 | | | 39.1 +/- 6.2 | | 0.589 | | |
|  | Plt (K cells/mL) | | | 249 +/- 78 | | | 226 +/- 61 | | | 260 +/- 85 | | 0.204 | | |
|  | | Neutrophils, absolute (k cells/mL) | | 5.94 +/- 5.67 | | | 4.79 +/- 1.59 | | | 6.57 +/- 6.92 | | 0.370 | | |
|  | | Lymphocytes, absolute (k cells/mL) | | 1.57 +/- 1.91 | | | 1.31 +/- 0.35 | | | 1.72 +/- 2.38 | | 0.549 | | |
|  | | Eosinophils, relative | | 2.3% +/- 2.0% | | | 2.2% +/- 1.7% | | | 2.4% +/- 2.1% | | 0.848 | | |
|  | | Eosinophils, absolute (k cells/mL) | | 0.15 +/- 0.14 | | | 0.16 +/- 0.15 | | | 0.15 +/- 0.14 | | 0.828 | | |
|  | | Elevated TTG IgA titer | | 1/15 (6.7%) | | | 0/9 (9.0%) | | | 1/6 (16.7%) | | 0.400 | | |
| Location of histopathologic inflammation | | | | | | | | | | | | | | |
|  | | Gastritis | | 4/38 (10.5%) | | | 0/13 (0.0%) | | | 4/25 (16.0%) | | Not calculated | | |
|  | | Enteritis | | 11/38 (28.9%) | | | 0/13 (0.0%) | | | 11/25 (44.0%) | | Not calculated | | |
|  | | Colitis | | 32/38 (84.2%) | | | 13/13 (100.0%) | | | 19/25 (76.0%) | | Not calculated | | |
| Hospital course outcomes | | | | | | | | | | | | | | |
| Admissions | | | | 13/38 (34.2%) | | | | 2/13 (15.4%) | | | 11/25 (44.0%) | | 0.148 | |
| Length of stay (days) | | | | | | | | | | | | | | |
|  | Mean +/- SD | | | 8.8 +/- 4.0 | | | | 7.5 +/- 2.1 | | | 9.1 +/- 4.3 | | 0.630 | |
|  | Median | | | 7.0 | | | | 7.5 | | | 7.0 | |  |  |
| Time from symptom onset to GEC symptom resolution (days) | | | | | | | | | | | | | | |
|  | | | Mean +/- SD | | 54.9 +/- 38.1 | 50.1 +/- 21.2 | | | 57.4 +/- 44.6 | | | | | 0.579 |
|  | | | Median | | 50.5 | 52.0 | | | 42.0 | | | | |  |
| Time from treatment to GEC symptom resolution (days) | | | | | | | | | | | | | | |
|  | | | Mean +/- SD | | 27.3 +/- 23.3 | 16.9 +/- 11.8 | | | 32.8 +/- 26.0 | | | | | **0.045*** |
|  | | | Median | | 21.0 | 14.0 | | | 23.0 | | | | |  |
| Absence of symptom recrudescence | | | | | | | | | | | | | | |
|  | At 1 month after initial resolution | | | 28/37 (75.7%) | | | | 10/12 (83.3%) | | | 18/25 (72.0%) | | 0.687 | |
|  | At 2 months after initial resolution | | | 28/34 (82.4%) | | | | 8/11 (72.7%) | | | 20/23 (87.0%) | | 0.363 | |
|  | At 3 months after initial resolution | | | 27/31 (87.1%) | | | | 9/11 (81.8%) | | | 18/20 (90.0%) | | 0.601 | |
|  | At 6 months after initial resolution | | | 20/24 (83.3%) | | | | 6/8 (75.0%) | | | 14/16 (87.5%) | | 0.578 | |
|  | At 12 months after initial resolution | | | 14/17 (82.4%) | | | | 4/6 (66.7%) | | | 10/11 (90.9%) | | 0.515 | |
| The p-value was calculated by ANOVA for numerical covariates and chi-square test or Fisher’s exact for categorical covariates, where appropriate. P-values for “Location of histopathologic inflammation” do not add significant meaning to the analysis, given the definitions of our groupings.  SD: standard deviation  IQR: interquartile range  ECOG: Eastern cooperative oncology group  CTCAE: common terminology criteria for adverse events  LDH: lactate dehydrogenase  GI: gastrointestinal  EGD: esophagogastroduodenoscopic | | | | | | | | | | | | | | |

**Supplemental Figure 1**

Kaplan-Meier survival curves for TTTF and PFS. ** denotes significance at α<0.05. (a) TTTF, stratified by colitis type. (b) PFS, stratified by colitis type. One patient’s clinical response to CPI therapy had not yet been evaluated at time of data collection.


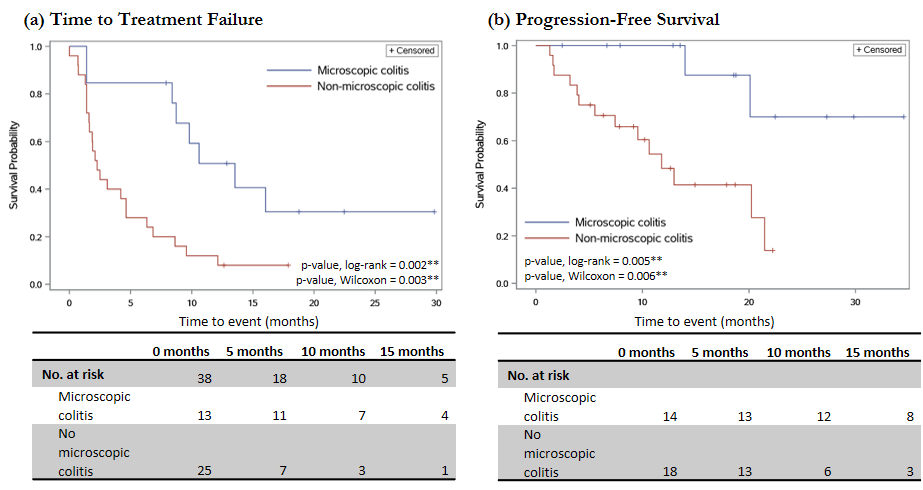

Supplement: Supplementary file 1 — Additional file 1: Table S1. Full listing of data collection variables determined a priori from clinical experience, sorted alphabetically. Table S2. Additional characteristics of prior irAEs. Prior irAEs defined as symptom manifestations of any adverse reaction felt related to CPI therapy, before onset of colitis. Any prior gastrointestinal irAEs that occurred while the patient was receiving a different CPI regimen. 3/38 (7.9%) patients had multiple prior irAEs. Table S3. Additional features and results characterizing patient presentations and clinical courses. Univariate analysis by colitis subset displayed. Enterocoltis symptoms were inquired after at standard oncologic follow-up visits. Of note, the total number of patients decreased over time, yielding decreasing denominators in “Absence of symptom recrudescence.” Figure S1. Kaplan-Meier survival curves for TTTF and PFS. ** denotes significance at α<0.05. (a) TTTF, stratified by colitis type. (b) PFS, stratified by colitis type. One patient’s clinical response to CPI therapy had not yet been evaluated at time of data collection. [file 40425_2019_756_MOESM1_ESM.docx]
